# Sensitivity and Specificity of Soluble Triggering Receptor Expressed on Myeloid Cells-1, Midregional Proatrial Natriuretic Peptide and Midregional Proadrenomedullin for Distinguishing Etiology and to Assess Severity in Community-Acquired Pneumonia

**DOI:** 10.1371/journal.pone.0163262

**Published:** 2016-11-15

**Authors:** Susanna Esposito, Maria Di Gangi, Fabio Cardinale, Eugenio Baraldi, Ilaria Corsini, Liviana Da Dalt, Pier Angelo Tovo, Antonio Correra, Alberto Villani, Oliviero Sacco, Laura Tenero, Piera Dones, Monia Gambino, Alberto Zampiero, Nicola Principi, for the Ita-CAP Study Group

**Affiliations:** 1 Pediatric Highly Intensive Care Unit, Department of Pathophysiology and Transplantation, Università degli Studi di Milano, Fondazione IRCCS Ca’ Granda Ospedale Maggiore Policlinico, Milan, Italy; 2 Pediatric Infectious Diseases Unit, G. Cristina Hospital, Palermo, Italy; 3 Pediatric Unit, Giovanni XXIII Hospital, Bari, Italy; 4 Pediatric Pulmonology Unit, Children’s Hospital, University of Padua, Padua, Italy; 5 Pediatric Emergency Unit, Policlinico Sant’Orsola, University of Bologna, Bologna, Italy; 6 Pediatric Unit, Treviso Hospital, University of Padua, Padua, Italy; 7 Pediatric Clinic, Regina Margherita Hospital, University of Turin, Turin, Italy; 8 Pediatric Unit, Santobono Hospital, Naples, Italy; 9 General Pediatrics and Infectious Diseases, IRCCS Bambino Gesù Hospital, Rome, Italy; 10 Pulmonology Unit, IRCCS Giannina Gaslini Hospital, Genoa, Italy; 11 Pediatric Clinic, University of Verona, Verona, Italy; Universite Paris Descartes, FRANCE

## Abstract

**Study Design:**

This study aimed to evaluate the diagnostic accuracy of soluble triggering receptor expressed on myeloid cells-1 (sTREM-1), midregional proatrial natriuretic peptide (MR-proANP) and midregional proadrenomedullin (MR-proADM) to distinguish bacterial from viral community-acquired pneumonia (CAP) and to identify severe cases in children hospitalized for radiologically confirmed CAP. Index test results were compared with those derived from routine diagnostic tests, i.e., white blood cell (WBC) counts, neutrophil percentages, and serum C-reactive protein (CRP) and procalcitonin (PCT) levels.

**Methods:**

This prospective, multicenter study was carried out in the most important children’s hospitals (n = 11) in Italy and 433 otherwise healthy children hospitalized for radiologically confirmed CAP were enrolled. Among cases for whom etiology could be determined, CAP was ascribed to bacteria in 235 (54.3%) children and to one or more viruses in 111 (25.6%) children. A total of 312 (72.2%) children had severe disease.

**Results:**

CRP and PCT had the best performances for both bacterial and viral CAP identification. The cut-off values with the highest combined sensitivity and specificity for the identification of bacterial and viral infections using CRP were ≥7.98 mg/L and ≤7.5 mg/L, respectively. When PCT was considered, the cut-off values with the highest combined sensitivity and specificity were ≥0.188 ng/mL for bacterial CAP and ≤0.07 ng/mL for viral CAP. For the identification of severe cases, the best results were obtained with evaluations of PCT and MR-proANP. However, in both cases, the biomarker cut-off with the highest combined sensitivity and specificity (≥0.093 ng/mL for PCT and ≥33.8 pmol/L for proANP) had a relatively good sensitivity (higher than 70%) but a limited specificity (of approximately 55%).

**Conclusions:**

This study indicates that in children with CAP, sTREM-1, MR-proANP, and MR-proADM blood levels have poor abilities to differentiate bacterial from viral diseases or to identify severe cases, highlighting that PCT maintains the main role at this regard.

## Introduction

Community-acquired pneumonia (CAP), with viruses and bacteria as its main causes, is one of the leading causes of morbidity and mortality in young children worldwide [1]. Early detection of bacterial cases that have the potential for a rapid negative evolution is essential to guide clinical management and to avoid prolonged hospitalization and the risk of death [2]. Furthermore, the differentiation of viral from bacterial CAP is necessary for the rational use of antibiotics and the consequent reduction in the emergence of bacterial resistance and drug-related adverse events [3]. Unfortunately, both these goals are difficult to achieve, particularly in younger children in whom the collection of respiratory samples is difficult or impossible to obtain [4]. Clinical signs and symptoms and radiological findings are frequently similar in cases of viral and bacterial disease [4]. Moreover, in most cases, the results from routine laboratory tests, such as white blood cell (WBC) count and C-reactive protein (CRP) serum level determination, tend to overlap, making the differentiation impossible [5]. This challenge also exists when using procalcitonin (PCT) to define the etiology and severity of CAP. PCT was the latest biomarker to enter into routine clinical practice [6].

These limitations explain why several attempts to find more effective biomarkers of CAP bacterial etiology and disease severity have been made in recent years. Recently, it has been suggested that soluble triggering receptor expressed on myeloid cells-1 (sTREM-1), midregional proatrial natriuretic peptide (MR-proANP) and midregional proadrenomedullin (MR-proADM) could improve the determination of CAP etiology and severity [7–9]. For all these biomarkers, data collected in adults seem to indicate that their concentrations in body fluids are increased in cases of bacterial infections, particularly in the most severe cases. However, the available data are limited and sometimes conflicting. Moreover, to date no evaluation was performed in children. This study aimed to evaluate the diagnostic accuracy of these new biomarkers to distinguish bacterial from viral CAP and to identify severe CAP cases in children. The results were compared with those derived from WBC counts, neutrophil percentages, and serum CRP and PCT levels.

## Materials and Methods

### Study design

This research was a prospective, multicenter study carried out in the 11 most important children’s hospitals of Italy (Fondazione IRCCS Ca’ Granda, Ospedale Maggiore Policlinico, Milan; Di Cristina Hospital, Palermo; Ospedale Giovanni XXIII, Bari; Padova Hospital, Padua; Ospedale Sant’Orsola, Bologna; Treviso Hospital, Treviso; Regina Margherita Hospital, Turin; Santobono Hospital, Naples; IRCCS Bambino Gesù Hospital, Rome; IRCCS Giannina Gaslini Hospital, Genoa, Italy; and Policlinico G.B. Rossi, Verona). The protocol was approved by the Ethics Committee of each center. Written informed consent was obtained from either the parent(s) or legal guardian(s) of each study participant, and children aged >8 years provided their written assent.

### Participants

Otherwise healthy children 4 months-14 years old consecutively hospitalized for clinical signs suggestive of CAP, such as tachypnea and abnormal breath sounds, and a radiological confirmation of CAP were recruited. Exclusion criteria included the presence of an underlying chronic disease or an antibiotic treatment of any type in the 48 hours before the admission. In each center, all chest radiographs were evaluated by an expert radiologist who classified the findings as alveolar CAP, non-alveolar CAP or no CAP in accordance with the World Health Organization (WHO) criteria for the standardized interpretation of pediatric chest radiographs for a diagnosis of pneumonia [10]. Chest radiography characterized by presence of consolidation (defined as a dense or fluffy opacity that occupies a portion or whole of a lobe or of the entire lung, that may or may not contain air-bronchograms above) or pleural effusion in the lateral pleural space was considered indicative of alveolar CAP. Non-alveolar CAP was diagnosed in case of linear and patchy densities (interstitial infiltrate) in a lacy pattern involving both lungs, featuring peribronchial thickening and multiple areas of atelectasis. The same diagnosis was made when minor patchy infiltrates not of sufficient magnitude to constitute primary consolidation and small areas of atelectasis that could not be distinguished from consolidation were evidenced. The CAP severity of disease was established in all the participating hospitals using the criteria indicated for children by the British Thoracic Society (BTS) [11]. In particular, features of severe disease in an infant were considered as follows: oxygen saturation <92%; cyanosis; respiratory rate >70 breaths/min; significant tachycardia for the fever level; prolonged central capillary refill time ≥2 s; difficulty in breathing; intermittent apnea; grunting; and not feeding. Features of severe disease in an older child included the following: oxygen saturation <92%; cyanosis; respiratory rate >50 breaths/min; significant tachycardia for the fever level; prolonged central capillary refill time ≥2 s; difficulty in breathing; grunting; and signs of dehydration. Both the evaluation of the chest radiograph and the classification of severity of each CAP episode were blinded to all the studied biological criteria, including WBC count, and CRP and PCT serum levels. After enrollment, within minutes from hospitalization the demographic, clinical history and clinical disease characteristics of each child were recorded. Moreover, a blood sample was drawn at admission to the hospital and divided in two parts: one sample was sent to the central laboratory of the hospital for the determination of routine tests including the WBC count, the percentage of neutrophils, and CRP level; the second sample was used for the determination of PCT, sTREM-1, MR-proANP, and MR-proADM levels as well as for pneumococcal and *Mycoplasma pneumoniae* detection. Finally, a nasopharyngeal swab was obtained from all the enrolled children using a pernasal nylon flocked swab and was stored in a tube of universal transport medium (Kit Cat. No. 360c, Copan Italia, Brescia, Italy) for respiratory virus, *Streptococcus pneumoniae*, and *Mycoplasma pneumoniae* detection. The serum of the blood samples that had to be used for new biomarkers’ serum level determination and nasopharyngeal samples were conserved in freezer at -80°C in each center and later sent to the laboratory of the Pediatric High Intensity Care Unit of the University of Milan for centralized processing.

### Test methods

#### Biomarker determination

WBC counts, neutrophil percentages and serum CRP levels were determined by the central laboratory of the hospital using routine methods. sTREM-1 concentrations were measured using an ELISA according to the manufacturer’s instructions (IQ Products, Groningen, the Netherlands) with a detection level of <7 pg/mL. An automated immunofluorescent assay was used for the determination of the levels of MR-proADM, MR-proANP and PCT according to the manufacturer’s instructions (BR·A·H·M·S, Germany). The functional assay sensitivity was previously assessed as being less than 0.25 nmol/L for MR-proADM, 10 pmol/L for MR-proANP, and 0.06 ng/mL for PCT. The detection limit that was calculated using the imprecision profile was previously assessed as being 0.02 ng/mL with a probability of 95% for PCT, 0.05 nmol/L for MR-proADM, and 2.1 pmol/L for MR-proANP. sTREM-1, MR-proADM, MR-proANP and PCT were chosen due to the sensitivity and specificity showed in adults with CAP for differentiating viral and bacterial CAP or severe and non-severe CAP [7–9]. Clinical and radiographic information, new biomarkers’ serum level determinations and results on nasopharyngeal samples were not available for the central laboratory of the hospital where routine methods were performed. Two different persons in the laboratory of the Pediatric High Intensity Care Unit of the University of Milan performed the determination of the new biomarkers and the viral and bacterial analyses on nasopharyngeal samples without exchanging information and in absence of any clinical and radiographic information.

#### Respiratory virus detection

Viral RNA or DNA was extracted from the respiratory secretions within 24 hours of collection using a Nuclisens EasyMAG automated extraction system (bioMérieux, Craponne, France) and was then tested using the Luminex x TAG respiratory virus panel fast assay (Luminex Molecular Diagnostics Inc., Toronto, Canada) to detect influenza A virus (subtype H1 or H3), influenza B virus, respiratory syncytial virus (RSV)-A and -B, parainfluenzavirus-1, -2, -3 and -4, adenovirus, human metapneumovirus (hMPV), coronaviruses 229E, NL63, OC43 and HKU1, enterovirus/rhinovirus (RV) and human bocavirus in accordance with the manufacturer’s instructions. The enterovirus/RV-positive samples were retested using a real-time polymerase chain reaction (PCR) assay using the iAg-Path-ID one step RT-PCR kit (Applied Biosystems, Foster City, CA) and the primers and probe sequences reported by Lu et al. to identify RV cases [12].

#### *Streptococcus pneumoniae* and *Mycoplasma pneumoniae* detection

To identify pneumococcal cases, nucleic acid extracts from blood and swab samples were tested for the autolysin-A (*LytA*) and wzg (*cpsA*) genes of *S*. *pneumoniae* using real-time PCR as previously described [13]. Each sample was tested in triplicate and was considered positive if at least 2 of the 3 tests were positive. To maximize sensitivity, no internal amplification control was used in the reaction, but there was an external control.

*M*. *pneumoniae* was looked for in blood and nasopharyngeal swabs with validated, nested PCR, as described previously [14].

#### Identification of probable bacterial and viral infection

Chest radiographs with alveolar or non-alveolar findings were initially classified as of possible bacterial or of possible viral origin, respectively, according to the WHO indication [10]. Then, radiological findings were coupled with results of the real-time PCR tests on blood samples and nasopharyngeal swabs. Evidence of *S*. *pneumoniae* or *M*. *pneumoniae* in these samples further supported bacterial etiology. Notably, *S*. *pneumoniae* can be detected in the nasopharyngeal secretions of children with viral CAP [15], but its presence in absence of viral detection in children with alveolar CAP is suggestive of probable pneumococcal CAP [16–18]. Moreover, even if it has been recently reported that *M*. *pneumoniae* is frequently carried in otherwise healthy children [19], it seems reasonable to think than when this pathogen is detected in children with CAP in absence of *S*. *pneumoniae* or respiratory viruses, it is the real cause of the lower respiratory infection independent of the radiological characteristics [20]. Finally, the presence of one or more respiratory viruses in the nasopharynx is commonly considered the etiologic agent of CAP because carriage of viruses in healthy subjects is uncommon [21]. In practical terms, CAP was considered to have a probable bacterial (PB) origin in the presence of 1) the detection of *S*. *pneumoniae* and *M*. *pneumoniae* in the blood with a chest radiograph indicative of any type of CAP; 2) a nasopharyngeal swab positive for *S*. *pneumoniae* associated with chest radiograph suggesting alveolar CAP; and 3) a nasopharyngeal swab positive for *M*. *pneumoniae* associated with chest radiograph suggesting any type of CAP. Probable viral (PV) CAP was diagnosed in the presence of a nasopharyngeal swab that was positive for one or more respiratory viruses associated with a chest radiograph leading to the diagnosis of non-alveolar CAP. Cases that could not be included in these groups were considered undetermined. Clinical information and blood test results were not available to the person that made this final classification of bacterial versus viral CAP:

### Analysis

A total sample size of 430 patients (assuming that about 60% of them have bacterial infection) achieves 80% power, with alpha = 0.05, to detect a change in sensitivity from 0.70 to 0.78 (and 83% power to detect a change in specificity from 0.7 to 0.8), using a two-sided binomial tests. Sample size was computed using PASS software v.11 (NCSS, LCC, Kaysville, Utah, USA).

All PB cases and all PV cases were evaluated together. Continuous variables are presented as the mean ± standard deviation (SD), and categorical variables are presented as numbers and percentages. Comparisons between groups (i.e., PB vs PV and severe vs non-severe CAP) were performed using the χ^2^ or Fisher’s exact test, as appropriate (for categorical variables), or a two-sided Student’s t-test after confirming that the data were normally distributed (based on the Shapiro-Wilk statistic) or a two-sided Wilcoxon’s rank-sum test otherwise (for continuous variables). Diagnostic performances of the biomarkers were evaluated with Receiver Operating Characteristic (ROC) curves and the area under ROC curve (AUC). The best cut-off values for different biomarkers were obtained based on the highest sensitivity and specificity through the roctab function in STATA. In case of indeterminate results for CRP and new biomarkers, the lowest limit of detection of the various methods was considered. Missing data were reported in the Tables and the missing information was not included in the statistical analyses. All analyses were conducted using SAS version 9.2 (Cary, NC, USA) and STATA version 11.0 (StataCorp LP, College Station, Tex) statistical packages.

## Results

### Participants

A total of 433 children (males, 56.0%; mean age 4.2 ± 3.5 years) with radiologically confirmed CAP were enrolled. Their demographic, clinical and laboratory characteristics are reported in **Table 1**. Results on respiratory viruses, *S*. *pneumoniae*, and *M*. *pneumoniae* were available for all the patients. CAP was ascribed to bacteria in 235 (54.3%) children and to one or more viruses in 111 (25.6%) children. In 87 cases (20.1%), the etiology of the disease was undetermined. Bacteremia was detected in 28 cases (6.5%): *S*. *pneumoniae* in 27 cases and *M*. *pneumoniae* one case. Globally, *S*. *pneumoniae* was considered the probable etiologic agent in 195 (83.0%) PB cases, and *M*. *pneumoniae* was considered the probable etiologic agent in 40 (17%) cases. Among PV CAP, RSV and RV were the most common and were detected as single pathogens in 52 (46.8%) and 12 (10.8%) children, respectively. In the remaining cases (47, 42.4%), co-infections between these viruses and other viral agents were found. Moreover, 312 (72.2%) children had severe disease. Among them, 178 (57.0%), 78 (25.0%), and 56 (18.0%) had a PB, a PV or an undetermined infection, respectively.

**Table 1:** Demographic, clinical, laboratory and radiographic variables in 433 children, according to their infection status*.

Variable	All subjects	Probable bacterial	Probableviral	Undetermined
	n = 433	n = 235	n = 111	n = 87
**Demographics and clinical presentation**				
Males (%)	242 (56.0)	140 (59.8) ^§^	53 (47.7) ^§^	49 (56.3)
Mean age ± SD (years)	4.2 ± 3.5	4.9 ± 3.6 ^§^^&^	2.8 ± 2.4 ^§^^ç^	3.9 ± 3.5 ^&^^ç^
Caucasians (%)	359 (85.7)	198 (87.2)	88 (83.0)	73 (84.9)
At least one parent smoked (%)	153 (36.9)	80 (35.6)	43 (40.6)	30 (35.7)
Presence of fever” (%)	287 (66.7)	173 (73.9) ^§^^&^	63 (57.3) ^§^	51 (59.3) ^&^
O_2_ therapy (%)	139 (33.3)	65 (28.4) ^§^	46 (44.2) ^§^	28 (33.3)
Clinical findings (%)				
Rhonchi	83 (19.3)	31 (13.2) ^§^	36 (32.4) ^§^^ç^	16 (18.6) ^ç^
Rales	351 (81.4)	186 (79.5) ^§^	98 (88.3) ^§^	67 (77.9)
Wheezing	104 (24.1)	45 (19.2) ^§^	34 (30.6) ^§^	25 (29.1)
Any of the above	365 (84.7)	192 (82.0) ^§^	103 (92.8) ^§^^ç^	70 (81.4) ^ç^
Severe disease	312 (72.2)	178 (75.7)	78 (70.3)	56 (65.1)
**Laboratory data**	**Mean ± SD (n)**	**Mean ± SD (n)**	**Mean ± SD (n)**	**Mean ± SD (n)**
WBC (cells/μL)	14213 ±8570 (428)	14776 ± 9104(232)	12523 ± 6436 (111)	14886 ± 9279 (85)
CRP, mg/L	16.7 ± 42.6 (426)	21.3 ± 48.1(232)^§^^&^	8.0 ± 30.4 (110)^§^^ç^	15.4 ± 38.7 (84)^&^^ç^
Neutrophils, %	61.2 ± 20.7 (413)	63.6 ± 20.7 (222)^§^	56.6 ± 19.7 (109)^§^	60.7 ± 21.2 (82)
PCT, ng/mL	4.1 ± 13.9 (265)	6.1 ± 17.0 (132) ^§^^&^	1.1 ± 3.4 (78)^§^	3.5 ± 14.4 (55)^&^
sTREM-1, pg/mL	95.7 ± 186.8 (405)	101.2 ± 193.1 (214)	82.5 ± 190.4 (108)	98.8 ± 165.7 (83)
MR-proANP, pmol/L ^a^	55.0 ± 48.6 (408)	53.0 ± 44.3 (222)	57.9 ± 56.2 (106)	56.8 ± 49.5 (80)
MR-proADM, nmol/L ^a^	0.44 ± 0.71 (410)	0.50 ± 0.94 (223) ^&^	0.37 ± 0.16 (106)	0.35 ± 0.17 (81)^&^

CRP, C-reactive protein; PCT, procalcitonin; SD, standard deviation; sTREM, soluble triggering receptor expressed on myeloid cells-1; MR-proANP, midregional proatrial natriuretic peptide; MR-proADM, midregional proadenomedullin; WBC, white blood cell count. Results on respiratory viruses, *Streptococcus pneumoniae*, and *Mycoplasma pneumoniae* were available for all the patients.

*The sums do not add up to the total because of missing values.

^**§**^p<0.05 for comparison between probable bacterial and probable viral infection groups.

^**&**^p<0.05 for comparison between probable bacterial and undetermined infection groups.

^**ç**^p<0.05 for comparison between probable viral and undetermined infection groups.

### Diagnostic performance of the studied biomarkers to predict CAP etiology

At admission, the percentage of neutrophils was significantly higher in children with PB than in those with PV CAP (63.6% ± 20.7 vs 56.6 ± 19.7; p<0.05). Similar results were observed for both CRP and PCT (CRP, 21.3 ± 48.1 mg/L in PB and 8.0 ± 30.4 mg/L in PV cases, p<0.05; PCT, 6.1 ± 17.0 ng/mL in PB and 1.1 ± 3.4 in PV CAP, p<0.05). Moreover, the levels of MR-proADM found in PB CAP cases were significantly higher than those in undetermined CAP cases (0.50 ± 0.94 vs 0.35 vs ± 0.17, p< 0.05).

For the evaluation of the diagnostic performance of the studied biomarkers, only children with defined PB or PV CAP were considered. Diagnostic performance of studied biomarkers at enrollment to predict bacterial and viral infections is reported in **Table 2**. All of them had low AUC values. However, CRP and PCT had the best performances for both PB (AUC of 0.66, 95% CI: 0.61–0.71, and 0.69, 95% CI: 0.63–0.75, respectively) and PV (AUC of 0.68, 95% CI: 0.62–0.63, and 0.67, 95% CI: 0.60–0.64, respectively) CAP identification. Cut-off values with the highest sensitivity and specificity combination for the identification of PB and PV infections using CRP were ≥7.98 mg/L and ≤7.5 mg/L, respectively. When PCT was considered, the cut-off values with the highest combined sensitivity and specificity were ≥0.188 ng/mL for PB CAP and ≤0.07 ng/mL for PV CAP. sTREM-1, MR-proANP, and MR-proADM had predictive values for both PB and PV infections that were lower than that evidenced for CRP and PCT but were not higher than that from the WBC count and neutrophil percentage, as evidenced by the AUC value that was lower than 0.60.

**Table 2:** Diagnostic performance of soluble triggering receptor expressed on myeloid cells-1 (sTREM), midregional proatrial natriuretic peptide (proANP) and midregional proadenomedullin (proADM) biomarkers, as compared to white blood cell (WBC) count, neutrophils percentage, C reactive protein (CRP) and procalcitonin (PCT) at enrolment to predict bacterial and viral infections, according to biomarker cut-off with highest sensitivity and specificity.

Variable	n	Biomarker cut-off value	Sensitivity	Specificity	PPV	NPV	AUC (95% CI)
**Probable bacterial infection (n = 235)**							
sTREM-1, pg/mL	405	≥69.56	31.8%	73.7%	58.1%	48.4%	0.50 (0.45–0.56)
MR-proANP, pmol/L*	408	≤59.1	76.1%	33.1%	58.3%	53.1%	0.52 (0.46–0.57)
MR-proADM, nmol/L	410	≥0.32	78.0%	35.7%	59.8%	57.0%	0.58 (0.52–0.63)
WBC count, cells/μL	428	≥12870	46.1%	61.3%	59.1%	48.3%	0.52 (0.47–0.58)
CRP mg/L	426	≥7.98	50.9%	80.4%	76.1%	57.1%	0.66 (0.61–0.71)
Neutrophils, %	413	≥61.0	63.5%	53.8%	62.1%	55.2%	0.58 (0.53–0.64)
PCT, ng/mL	265	≥0.188	67.4%	65.1%	66.4%	66.1%	0.69 (0.63–0.75)
**Probable viral infection (n = 111)**							
sTREM-1, pg/mL*	405	≤59.28	72.2%	36.4%	29.4%	78.1%	0.52 (0.46–0.58)
MR-proANP, pmol/L*	408	≤35.5	46.2%	61.5%	29.9%	76.3%	0.51 (0.44–0.57)
MR-proADM, nmol/L*	410	≤0.31	35.8%	73.4%	32.2%	76.5%	0.53 (0.46–0.59)
WBC count, cells/μL*	428	≤15740	78.4%	33.1%	29.3%	81.2%	0.56 (0.50–0.62)
CRP, mg/L*	426	≤7.5	88.2%	46.3%	36.6%	91.8%	0.68 (0.62–0.73)
Neutrophils, %*	413	≤60.8	56.9%	60.1%	34.1%	79.4%	0.60 (0.54–0.66)
PCT, ng/mL*	265	≤0.07	48.7%	81.1%	52.0%	78.9%	0.67 (0.60–0.74)

AUC, area under the curve; 95% CI, 95% confidence interval; NPV, negative predictive value; PPV, positive predictive value.

*Inverse relationship between biomarker and probable bacterial/viral infection.

### Diagnostic performance of studied biomarkers to predict severity of CAP

**Table 3**shows the biomarker levels at enrollment according to the severity of the disease. All the studied parameters, with the exception of MR-proADM, were significantly higher in severe CAP compared with non-severe CAP (p<0.01 for MR-proANP, WBC count, neutrophil percentage and PCT; p<0.05 for sTREM-1 and CRP).

**Table 3:** Biomarkers at enrolment according to the severity of disease^&^.

	Severe disease (n = 312)		Non-severe disease (n = 120)	
Biomarker	n	Mean ± SD	n	Mean ± SD
sTREM-1, pg/mL	290	100.3 ± 198.3 ^§^	114	84.8 ± 154.8 ^§^
MR-proANP, pmol/L	292	57.8 ± 46.4 *	115	45.4 ± 44.8 *
MR-proADM, nmol/L	294	0.42 ± 0.38	115	0.48 ± 1.19
WBC count, cells/μL	309	14763 ± 8552 *	119	12786 ± 8489 *
CRP, mg/L	307	17.9 ± 45.6 ^§^	119	13.7 ± 33.7 ^§^
Neutrophils, %	296	62.9 ± 20.4 *	117	56.7 ± 20.7 *
PCT, ng/mL	194	4.6 ± 15.3 *	71	2.6 ± 9.1 *

CRP, C-reactive protein; PCT, procalcitonin; SD, standard deviation; sTREM, soluble triggering receptor expressed on myeloid cells-1; MR-proANP, midregional proatrial natriuretic peptide; MR-proADM, midregional proadenomedullin; SD, standard deviation; WBC, white blood cell count.

^&^ One subject could not be categorized as severe or non-severe disease due to missing data.

^**§**^ p<0.05 for comparison between severe and non-severe disease groups.

* p<0.01 for comparison between severe and non-severe disease groups.

Even for the identification of severe cases, the predictive value of all the studied biomarkers was poor (**Table 4**). The best results were obtained when PCT (AUC = 0.65, 95% CI: 0.57–0.63) and MR-proANP (AUC = 0.65, 95% CI: 0.59–0.71) were evaluated. However, in both cases, the biomarker cut-off with the highest combined sensitivity and specificity (≥0.093 ng/mL for PCT and ≥33.8 pmol/L for proANP) had relatively good sensitivity (higher than 70%) but limited specificity (of approximately 55%). Other biomarkers were even less effective (all AUC < 0.60).

**Table 4:** Diagnostic performance of soluble triggering receptor expressed on myeloid cells-1 (sTREM), midregional proatrial natriuretic peptide (proANP) and midregional proadenomedullin (proADM) biomarkers, as compared to white blood cell (WBC) count, neutrophils percentage, C reactive protein (CRP) and procalcitonin (PCT) at enrolment to predict severity of disease, according to biomarker cutoff with highest sensitivity and specificity.

Variable n	Biomarker cut-off value	Sensitivity	Specificity	PPV	NPV	AUC (95% CI)
Severe disease (n = 312)						
sTREM-1, pg/mL 404	≥16.32	77.2%	38.6%	76.2%	40.0%	0.57 (0.51–0.64)
MR-proANP, pmol/L 407	≥33.8	70.9%	56.5%	80.5%	43.3%	0.65 (0.59–0.71)
MR-proADM, nmol/L 409	≥0.39	51.4%	66.1%	79.5%	34.7%	0.55 (0.49–0.61)
WBC count, cells/μL 428	≥13240	44.7%	71.4%	80.2%	33.2%	0.59 (0.53–0.65)
CRP, mg/L 426	≥1.04	77.2%	41.2%	77.2%	41.2%	0.58 (0.52–0.64)
Neutrophils % 413	≥55.4	69.9%	50.4%	78.1%	39.9%	0.59 (0.53–0.65)
PCT, ng/mL 265	≥0.093	76.8%	57.7%	83.2%	47.7%	0.65 (0.57–0.73)

AUC, area under the curve; 95% CI, 95% confidence interval; NPV, negative predictive value; PPV, positive predictive value.

## Discussion

This study is the first to evaluate the utility of serum sTREM-1, MR-proANP, and MR-proADM concentrations in predicting the etiology and severity of pediatric CAP in comparison to routine biomarkers. In this study, to overcome the clinical, radiological and laboratory problems that limit the definition of etiology and severity of CAP in children, the identification of bacterial and viral CAP was based on the criteria usually accepted by the international literature [16–18]. A combined evaluation of radiological findings and detection in the blood and in nasopharyngeal samples of the most important respiratory viral and bacterial agents of CAP in children, was performed. Moreover, to establish severity, the criteria suggested by BTS were used. Despite a certain number of CAP cases, probably those due to mixed infection, remained undetermined, this method has probably lead to the identification of those CAPs which are more likely due only to bacteria or only to viruses. Interestingly, as reported in several recent studies and probably thanks to the new molecular diagnostic methods that permit us to enlarge in comparison with the past possibilities for viral identification [4], the prevalence of PB and PV among children with severe CAP was similar. A global evaluation of the results of this study seemed to indicate that in children with CAP, sTREM-1, MR-proANP, and MR-proADM blood levels are unable to differentiate bacterial from viral diseases or to identify severe cases. At admission, the mean values of all these biomarkers were similar in PB and PV cases. When attempts to evaluate the sensitivity and specificity of each of these biomarkers in defining the etiology and severity of the studied CAP cases were made, either the sensitivity or the specificity was found to be very low, leading to a modest predictive value. This result was confirmed by the values of the AUC, which were always below 0.70 for a single biomarker for both the definition of the etiology and the assessment of severity, a value that suggests poor accuracy of the studied tests. Moreover, for the definition of the etiology of pediatric CAP, the predictive ability of these biomarkers seemed to be lower than that of CRP and PCT, whereas for the identification of severe cases the best results were obtained with evaluations of PCT and MR-proANP.

The diagnostic relevance of sTREM-1 in bacterial diseases, including CAP, has been studied in experimental animal studies and in adult humans with conflicting results. Some studies have reported that in cases of sepsis or CAP, sTREM-1 is a superior indicator of bacterial disease compared with CRP and PCT [22, 23]. Moreover, it was demonstrated that sTREM-1 levels were significantly higher in neonates with sepsis than in healthy controls [24]. Finally, in children with bronchiectasis, sTREM-1 sputum levels correlated with markers of neutrophilic inflammation but not necessarily with CRP concentrations, suggesting that sTREM-1 may be more sensitive in detecting pulmonary neutrophilic inflammation than CRP [25]. However, other studies have reported results similar to those found by our study. In adults with ventilator-associated pneumonia, sTREM1 was a poor predictor of VAP among critically ill subjects undergoing direct bronchoscopy [26, 27]. Different patient and sample characteristics used to measure sTREM-1 concentrations might explain the different results. In most of the cases showing a role for sTREM-1 in the diagnosis of bacterial infection, the data were collected in patients with a chronic underlying disease who were hospitalized in intensive care unit, whereas in this study, only otherwise healthy children with an acute CAP episode were enrolled. Moreover, the different criteria used to classify the etiology and severity of CAP could explain the differences among the results presented in the literature.

Changes in MR-proANP have been associated with acute and chronic heart failure [28], although the prognostic value of this biomarker in CAP seems to be maintained independent of chronic heart failure [29]. Moreover, in diseases associated with bacteremia, this biomarker is increased [30]. Because CAP with bacteremia is generally the most severe, it was concluded that this biomarker could simultaneously detect bacterial CAP and identify the most severe cases. Alan et al. reported that the addition of blood biomarkers, including MR-proANP, to clinical scores significantly improved the prognostic capabilities of the pneumonia severity index [31]. Kruger et al. reported that MR-proANP was a good predictor of mortality risk in patients with CAP [29]. However, the poor ability by MR-proANP to predict bacteremia in patients with CAP was evidenced by Guinard-Barbier et al. [32]. Additionally, in this case, differences in the characteristics of the studied patients might explain the results. None of the children in our study had chronic heart failure, and the number of bacteremia cases was very small. It is possible that the blood levels of this biomarker significantly increased and had a true prognostic value only in very severe bacteremic cases of CAP. Moreover, the criteria used in this study to measure severity were different from those used in the studies in which adults were enrolled. In those studies, severity was measured considering the final outcome of the disease, whereas in this study, consistent with pediatric guidelines, severity was considered only at admission.

Similar conclusions can be drawn for the MR-proADM evaluation. Additionally, this marker has been tested mainly as a marker of severity, although there are data indicating that it can be of value in the diagnosis and prognosis of sepsis and bacterial CAP [9]. Once again, it seems highly likely that modifications of this biomarker occur only in very severe bacterial diseases that are relatively uncommon iamong children with CAP.

The problems that pediatricians have in differentiating bacterial from viral CAP and in assessing the severity of the disease are highlighted by the poor predictive value evidenced in this study for WBC count, neutrophil percentage, and CRP and PCT levels. CRP and PCT were slightly better than WBC count and neutrophil percentage. However, the importance of CRP and PCT in the definition of the etiology and the severity of pediatric CAP has been largely studied and discussed with conflicting results. CRP as a type of acute-phase reaction protein is closely related with inflammatory reaction and tissue injuries and can be influenced by factors other than bacterial components. For many years, it has been shown that only extremely high CRP serum levels are associated with bacterial disease and a negative prognosis, whereas in many cases, such values do not permit an estimate of the real etiology of the disease [32–34]. Cohen et al. showed that PCT and not CRP was the only independent predictor of apyrexia in children hospitalized for CAP [35]. However, recently, it has been reported that CRP can predict outcomes of pediatric CAP cases because CRP levels are significantly associated with both fever duration and hospital length of stay [36]. For every 1 mg/dL increase in CRP, the length of stay increased by 1 hour. Moreover, it was shown that extremely elevated CRP levels are associated with unfavorable outcomes, including death, in pediatric patients, highlighting the importance of very high levels, uncommonly assessed in daily practice [37]. Similarly, the results reported for PCT are inconsistent. Nascimento-Carvalho et al. showed that in 95 children, PCT had a negative predictive value for differentiating bacteremic infections from viral infections, atypical bacterial infections, and nonbacteremic typical bacterial infections [38]. More recently, Galetto-Lacour et al. reported a positive role of PCT in differentiating bacterial from viral CP, particularly when the disease was due to *S*. *pneumoniae* [39]. Additionally, we previously used a PCT cut-off value to identify PV CAP and to guide antibiotic therapy [40]. Conflicting results were reported in other studies. Korpi et al. reported that PCR had a sensitivity in the identification of bacterial CAP of only 55% [41], whereas later, these authors found that high values of PCT ≥5 ng/mL were associated with a high predictivity of bacterial CAP [42]. However, it seems evident that only very high serum concentrations of PCT are predictive of true bacterial CAP. Regarding PCT and disease severity, a study by Don et al. found that higher mean values were present in children who needed hospitalization; additionally, in this case, a non-marginal number of cases could not be classified because of similar results among children with mild and severe disease [43].

Limitations of our study include the missing data for laboratory biomarkers in some patients, potential classification bias in the etiologic diagnosis, and the fact that patients with undetermined infection were excluded from the analysis. However, our evaluation has been done in a large study population with an appropriate calculation of sample size even excluding patients with undetermined infection. In addition, viral and bacterial results in nasopharyngeal aspirates and blood were available for all the study patients. Moreover, a sophisticated statistical analyses has been performed, although further research on the role of the studied biomarkers in CAP and other infectious diseases is recommended.

In conclusion, most of the problems related to the evaluation of the PB or PV origin of a pediatric CAP case together with the prevision of its outcome remain unsolved. No advantages are given by the use of sTREM-1, MR-proANP, and MR-proADM, whereas PCT maintains its role. More studies are needed to assure a proper approach to CAP therapy in children and to avoid the risk of useless antibiotic therapy.
